# Mitochondrial Transcription Factor A, an Endogenous Danger Signal, Promotes TNFα Release via RAGE- and TLR9-Responsive Plasmacytoid Dendritic Cells

**DOI:** 10.1371/journal.pone.0072354

**Published:** 2013-08-12

**Authors:** Mark W. Julian, Guohong Shao, Zachary C. VanGundy, Tracey L. Papenfuss, Elliott D. Crouser

**Affiliations:** 1 Dorothy M. Davis Heart and Lung Research Institute, Division of Pulmonary, Allergy, Critical Care, and Sleep Medicine, Wexner Medical Center, the Ohio State University, Columbus, Ohio, United States of America; 2 College of Veterinary Medicine, Department of Veterinary Biosciences, the Ohio State University, Columbus, Ohio, United States of America; University of Medicine and Dentistry of New Jersey - New Jersey Medical School, United States of America

## Abstract

**Objective:**

Mitochondrial transcription factor A (TFAM) is normally bound to and remains associated with mitochondrial DNA (mtDNA) when released from damaged cells. We hypothesized that TFAM, bound to mtDNA (or equivalent CpG-enriched DNA), amplifies TNFα release from TLR9-expressing plasmacytoid dendritic cells (pDCs) by engaging RAGE.

**Materials and Methods:**

Murine Flt3 ligand-expanded splenocytes obtained from C57BL/6 mice were treated with recombinant human TFAM, alone or in combination with CpG-enriched DNA with subsequent TNFα release measured by ELISA. The role of RAGE was determined by pre-treatment with soluble RAGE or heparin or by employing matching RAGE (-/-) splenocytes. TLR9 signaling was evaluated using a specific TLR9-blocking oligonucleotide and by inhibiting endosomal processing, PI3K and NF-κB. Additional studies examined whether heparin sulfate moieties or endothelin converting enzyme-1 (ECE-1)-dependent recycling of endosomal receptors were required for TFAM and CpG DNA recognition.

**Main Results:**

TFAM augmented splenocyte TNFα release in response to CpGA DNA, which was strongly dependent upon pDCs and regulated by RAGE and TLR9 receptors. Putative TLR9 signaling pathways, including endosomal acidification and signaling through PI3K and NF-κB, were essential for splenocyte TNFα release in response to TFAM+CpGA DNA. Interestingly, TNFα release depended upon endothelin converting enzyme (ECE)-1, which cleaves and presumably activates TLR9 within endosomes. Recognition of the TFAM-CpGA DNA complex was dependent upon heparin sulfate moieties, and recombinant TFAM Box 1 and Box 2 proteins were equivalent in terms of augmenting TNFα release.

**Conclusions:**

TFAM promoted TNFα release in a splenocyte culture model representing complex cell-cell interactions *in vivo* with pDCs playing a critical role. To our knowledge, this study is the first to incriminate ECE-1-dependent endosomal cleavage of TLR9 as a critical step in the signaling pathway leading to TNFα release. These findings, and others reported herein, significantly advance our understanding of sterile immune responses triggered by mitochondrial danger signals.

## Introduction

Organ failures occurring in the context of critical illness are a leading cause of death in humans. With appropriate initial treatments and supportive care, many patients survive the early phases of critical illness, and most of the mortality occurs several days after the initial insult. This poorly understood complication of critical illness, frequently referred to as the multiple organ dysfunction syndrome (MODS), is characterized by the insidious loss of vital and non-vital (e.g., skeletal muscle) organ functions and is associated with signs of ongoing systemic and/or local inflammation (e.g., fever, elevated white blood cell counts, and neutrophilic infiltration of tissues). The latter has been referred to as “sterile inflammation” as no evidence of active infection can be identified. The cause of MODS is poorly understood, and the current treatment of these patients is restricted to supportive care or attempts to compensate for impaired organ function (e.g., mechanical ventilators, hemodialysis). However, recent studies have identified mitochondrial danger signals as critical mediators of sterile inflammation [1–4].

Mammalian immune systems have evolved to sense danger arising from the environment in the forms of potentially pathogenic infections or from internal sources, including malignantly transformed or functionally disabled (e.g., senescent or non-viable) cells. Immunogenic “danger signals” originating from the environment and host share common epitopes or biochemical characteristics that are detected by various pattern-recognizing receptors, including highly conserved Toll-like receptors (TLR). Sterile inflammation following acute cell and tissue damage has been linked to the release of otherwise concealed antigens derived from mitochondria. In particular, mitochondrial DNA (mtDNA) is shown to be sensed by TLR9 to promote systemic inflammation and organ damage [1]. However, the mechanisms linking TLR9, which in humans is expressed in specialized cell types, to sterile inflammation remain unclear.

Plasmacytoid dendritic cells (pDCs) are highly efficient antigen-presenting cells specialized for detection of immunogenic CpG-enriched DNA to produce Type I interferons (IFNs), a class of cytokines required for effective viral clearance by the host but of unclear relevance in the context of sterile inflammation [5]. Recent studies in our laboratory indicate that sensing of CpG-enriched DNA by TLR9-expressing pDCs is enhanced by mitochondrial transcription factor A (TFAM), a ubiquitous mitochondrial DNA-binding protein, to produce Type I IFNs [2]. However, pDCs can simultaneously produce TNFα, albeit through an alternate intracellular signaling pathway [6], and each pDC is capable of activating many adjacent immune cells [7]. In this regard, as pDCs represent a small fraction of the immune cell population in tissues, the biological consequences of pDC activation are primarily related to the stimulation of other immune cells, a phenomenon referred to as “bystander” activation [8]. Thus, in the context of sterile immune responses, we hypothesized that TFAM would amplify TNFα release in response to immunogenic DNA in a representative immune organ (the spleen) and that pDCs would contribute significantly to the production of TNFα under these sterile conditions. Furthermore, we sought to clarify whether the signaling mechanisms for Type I IFN production in response to TFAM-CpGA DNA complexes, which were previously defined [2], also regulate proinflammatory cytokine (TNFα) release.

## Materials and Methods

### Ethics Statement

All experiments were approved by The Ohio State University Institutional Laboratory Animal Care and Use Committee, and all care and handling of the animals were in accord with National Institutes of Health guidelines.

### Reagents

Murine CpG-containing oligonucleotide, type A (CpGA, TLR9 ligand), the guanosine-rich inhibitory oligonucleotide (G-ODN, TLR9 signaling inhibitor), and an inhibitor of endosomal nucleic acid binding (chloroquine) were obtained from InvivoGen (San Diego, CA). Cyclosporin H (CsH), Boc-Phe-Leu-Phe-Leu-Phe (Boc-FLFLF, BOC) and WRW4 (Trp–Arg–Trp–Trp–Trp–Trp) were acquired from Axxora, LLC (San Diego, CA), ChemPep, Inc. (Miami, FL) and Tocris Bioscience (Bristol, UK) respectively. Human *N*-formyl peptide [fMMYALF, the N-terminal sequence of mitochondrial NADH dehydrogenase subunit 6 (ND6)] was synthesized by GenScript Corp. (Piscataway, NJ). Bacterial *N*-formyl-Met-Leu-Phe (fMLP), Heparin lyases (Heparinases) II and III, along with endothelin converting enzyme-1 (ECE-1) inhibitors (SM-19712, phosphoramidon), and an endosomal acidification inhibitor (Bafilomycin A1) were obtained from Sigma-Aldrich Corp. (St. Louis, MO). The phosphoinositide 3-kinase (PI3K) inhibitor, LY294002, was acquired from Cell Signaling Technology, Inc. (Danvers, MA); BAY 11-7085 and Heparin sodium were obtained from Calbiochem® (EMD Biosciences, Inc., Rockland, MA) and APP Pharmaceuticals, Inc. (Schaumburg, IL) correspondingly. Primary antibodies were acquired commercially: TLR9 and β-actin (Santa Cruz Biotechnology, Inc., Santa Cruz, CA) and p-Akt, p-ERK and p-NF-κB (Cell Signaling Technology, Inc.). Anti-human TFAM polyclonal antibody was made by Spring Valley Laboratories, Inc. (Woodbine, MD). The epitope used to create the antibody was KQRKYG, which was chosen because the corresponding peptide sequence was unique relative to that of high mobility group box protein 1 (HMGB1). Secondary antibodies were obtained from Cell Signaling Technology, Inc. Recombinant human ECE-1 (R&D Systems, Inc., Minneapolis, MN) and recombinant human TLR9 (Abnova Corp., Taipei, Taiwan) were acquired for *in vitro* experimental use. HepG2 and HEK-293 cells were purchased from the American Type Culture Collection (ATCC; Manassas, VA) and cultured using DMEM/F-12 and MEM media (Life Technologies, Grand Island, NY), respectively. Unless otherwise stated, all additional chemicals were obtained from Sigma-Aldrich Corp. using the best available grade.

### Plasmid Construction and Purification of Recombinant Proteins

Recombinant mitochondrial transcription factor A (TFAM) was synthesized as described in detail previously [3]. In brief, the coding region of human TFAM was amplified by PCR, cloned into pcDNA3.1(-)/*myc*-His A (Life Technologies) vector in-frame or with added C-terminal *myc* epitope and 6×histidine tags and then transformed into *E. coli*-competent cells (DH5α, Life Technologies). The resultant TFAM plasmid, pcDNA3.1-TFAM. *myc*.6× His, was transfected into HEK-293 cells, and the resultant polyhistidine-tagged recombinant protein (TFAM) was then purified by Ni-NTA nickel-chelating resin. An entirely similar step-by-step process was used to create the recombinant DNA-binding TFAM Box 1 and TFAM Box 2 proteins [3]. As in the PCR amplification used to generate full-length recombinant TFAM, the sense primers for TFAM Box 1 (5’-CCGAATTCCCACCATGTCATCTGTCTTGGCAAGT-3’) and TFAM Box 2 (5’-GGGAATTCCCACCATGCTTGGAAAACCAAAAAGACCT-3’) introduced a consensus Kozak translation initiation sequence and an EcoRI restriction site to facilitate cloning. The anti-sense primers of TFAM Box 1 (5’-GGAAGCTTTTTTCCAAGCAGTGTTAACTC-3’) and TFAM Box 2 (5’-CCAAGCTTTTCATCATCATCATCTTCTTCT-3’) introduced a HindIII restriction site. Likewise, the coding region for human receptor for advanced glycation endproducts (hRAGE) comprising only the extracellular domain was cloned into the pcDNA3.1(-)/*myc*-His A vector and processed similarly to obtain recombinant soluble RAGE (sRAGE) as formerly detailed [2]. The identity and function of the recombinant proteins were confirmed by Western blot, capillary-liquid chromatography-nanospray tandem mass spectrometry (Nano-LC/MS/MS) and the electrophoretic mobility shift assay [2,3].

### HepG2 Total Mitochondrial Protein Fraction

HepG2 cell necrosis was induced by freeze/thaw and was confirmed by the lactate dehydrogenase (LDH) assay, as described previously [2,3,9]. The total mitochondrial protein cellular subfraction was prepared from these HepG2 cells using a differential centrifugation approach [10] with minor modifications. The endotoxin level was confirmed to be <5 pg/µg protein, and the absence of *Mycoplasma* contamination was established as detailed earlier [2,3].

### Mouse pDC Expansion and Splenocyte Preparation

Eight-to-ten-week old, adult, male, C57BL/6 mice (~25 g) (The Jackson Laboratory, Bar Harbor, ME) were employed for *in vivo* pDC expansion and the subsequent source for all splenocytes. pDC expansion was carried out using melanoma cells [expressing murine (Flt3)] as previously described [11,12]. Briefly, 4 × 10^6^ B16 melanoma cells (C57BL/6 background), transfected with mouse recombinant Flt3 ligand (Flt3L) cDNA using an MFG-retroviral vector, were suspended in sterile saline and injected subcutaneously in two sites over both flanks. Mice were euthanized when the tumor size reached 1-1.5 cm in diameter (after 3-4 weeks), and the spleens were harvested.

Single cell splenocyte suspensions were prepared following mechanical disaggregation of the spleen tissue and gently passing the released cells and tissue fragments through a 70 µm nylon cell strainer. Following erythrocyte lysis, cells were washed and then suspended in RPMI 1640 (supplemented with 25 mM HEPES, 2 mM L-glutamine, 50 U/ml penicillin, 50 µg/ml streptomycin, and 10% FBS) (Life Technologies). Cells were then cultured in 48-well plates at a concentration of 1 × 10^6^/ml in the above media with only 2% FBS at 37° C in a 5% CO_2_-humidified atmosphere. Polymyxin B (10 µg/ml) was routinely added to the cells in each well to further block the effect of any possible endotoxin contamination. After 1 hour, desired concentrations of total mitochondrial protein (5 µg/ml), or murine CpGA DNA (0.3 µM) ± TFAM (5 µg/ml, or similarly, TFAM Box 1 or TFAM Box 2) or immunoprecipitated TFAM-mtDNA [IP TFAM (0.5 µg/ml), see below] were then added to the medium. All pre-incubations with inhibitors of RAGE (sRAGE, 20 µg/ml; Heparin sodium, 1-100 U/ml), TLR9 (G-ODN, 10 µM), formyl peptide receptor (FPR) (CsH, 1 µM; BOC, 10 µM; WRW4, 20 µg/ml), endosomal acidification (chloroquine, 100 µM; Bafilomycin A1, 1 µM), PI3K signaling (LY294002, 5 µM), NF-κB signaling (BAY 11-7085, 5 µM) or ECE-1 (SM-19712, 10 µM; phosphoramidon, 100 µM) or FPR agonists (fMLP, 5 µg/ml; ND6, 5 µg/ml) or Heparin lyases II or III (5 mU/ml) were made as a one-time dose 30 minutes prior to TFAM + CpGA DNA addition. The cell supernatants were then collected at ~24 hours post-treatment and stored at -80° C for later analyses. Similar experiments involving treatment with TFAM + CpGA DNA or IP TFAM were carried out in Flt3L-expanded mouse splenocytes isolated from matching RAGE knockout (RAGE -/-) mice (generously provided by Angelika Bierhaus, Ph.D. and Peter P. Nawroth, M.D.) wherein RAGE depletion was confirmed by real-time PCR [2].

Elevated pDC populations following Flt3L expansion were identified via flow cytometry following immunofluorescent staining of splenocytes with mouse anti-plasmacytoid dendritic cell antigen 1 (mPDCA-1)-FITC (Miltenyi Biotec, Inc., Auburn, CA) [2]. The Mouse Plasmacytoid Dendritic Cell Isolation Kit II (Miltenyi Biotec, Inc.) was employed according to the manufacturer’s recommendations, when needed, to remove the pDCs from the splenocyte preparations. These isolated pDCs were then cultured similarly in 96-well plates and employed for additional select treatment experiments as presented.

### Tumor Necrosis Factor alpha (TNFα) Measurements

Mouse splenocyte supernatants, collected ~24 hours post-treatment, were analyzed for their TNFα concentrations by ELISA (eBioscience, Inc., San Diego, CA) according to the manufacturer’s recommendations.

### Immunoprecipitation of Native TFAM

Necrotic HepG2 cell (~7 × 10^6^/ml) lysates induced by freeze/thaw [2–4] underwent immunoprecipitation (IP) designed to pull down native TFAM protein (IP TFAM) [2]. After the addition of protease inhibitors [1 mM phenylmethylsulfonyl fluoride (PMSF), 10 µl/ml protease inhibitor cocktail (Sigma-Aldrich Corp.)] and centrifuging at 1500 × *g* for 10 min at 4^°^ C to remove cell debris, lysate supernatant was cleared of native immunoglobulins by the addition of ~30 µl/ml Protein A/G PLUS-Agarose (Santa Cruz Biotechnology, Inc.) with rotary agitation for 1 hour at 4^°^ C. Following centrifugation (2000 rpm for 5 min), the supernatant was incubated with rabbit anti-human TFAM antibody (1 µg/100 µg IP protein) under rotary agitation overnight at 4^°^ C. The next day, the supernatant was incubated with ~30 µl/ml Protein A/G PLUS-Agarose under rotary agitation for 4 hours at 4^°^ C. The agarose beads were then pelleted as before, washed with PBS several times and re-suspended in 100 µl of PBS with added protease inhibitor. After heating at 100^°^ C for 5 min, agarose beads were pelleted as before, and the resultant supernatant stored at -80^°^ C. The presence and source of DNA associated with the IP protein was determined by PCR and the presence of TFAM confirmed by Western blot as detailed previously [2].

### Signaling in Mouse Splenocytes

PI3K (i.e., Akt, ERK) and NF-κB signaling in mouse splenocytes was investigated following their treatment with CpGA DNA (0.3 µM) alone or in combination with TFAM (5 µg/ml). Cells were collected at 15, 30 and 60 minutes post-treatment and processed for Western blot analyses according to standard techniques. Possible inhibition of these signaling pathways was further examined by pre-treatment with sRAGE (20 µg/ml), LY294002 (5 µM) or BAY 11-7085 (5 µM) for 30 minutes. The rabbit polyclonal primary antibodies employed were p-Akt (1:500), p-ERK (1:1000) and p-NF-κB (1:1000). Protein band densities were normalized to protein load established by subsequent use of mouse monoclonal β-actin antibody (1:5000) and compared to corresponding bands from untreated cells.

### In Vitro Analysis of TLR9 Degradation by ECE-1

Recombinant human TLR9 (50 µM) was incubated with recombinant human ECE-1 (0.3 µg/ml) in an assay buffer (0.1 M MES, 0.1 NaCl) at the optimal pH for the enzyme (6.0) and at normal pH (7.4) in compliance with manufacturer recommendations for 5, 15, 30 and 60 minutes. Collected samples were then processed for Western blot analysis according to standard techniques employing a mouse monoclonal primary antibody to TLR9 (1:1000). Protein band densities from assayed samples were compared to corresponding bands from untreated TLR9 samples.

### Statistical Analyses

The data was derived from independent experiments, as designated in the figure legends, and was expressed as mean ± SEM, and statistical significance was based upon a value of *p* ≤ 0.05. SigmaPlot 12.0 and SYSTAT 13.0 software were used to plot the data and carry out the statistical analyses, respectively. Splenocyte TNFα release in response to various treatments was compared to untreated controls using the Wilcoxon Rank-Sum test. Additional comparisons involving pre-treatment inhibition studies and all experiments relating multiple groups were made using the Kruskal-Wallis test. Where appropriate, *post hoc* analyses between group rank means were performed using Dunn’s test.

## Results

### Splenocyte TNFα release in response to CpGA DNA was amplified by TFAM and was promoted by pDCs

**Figure 1:**
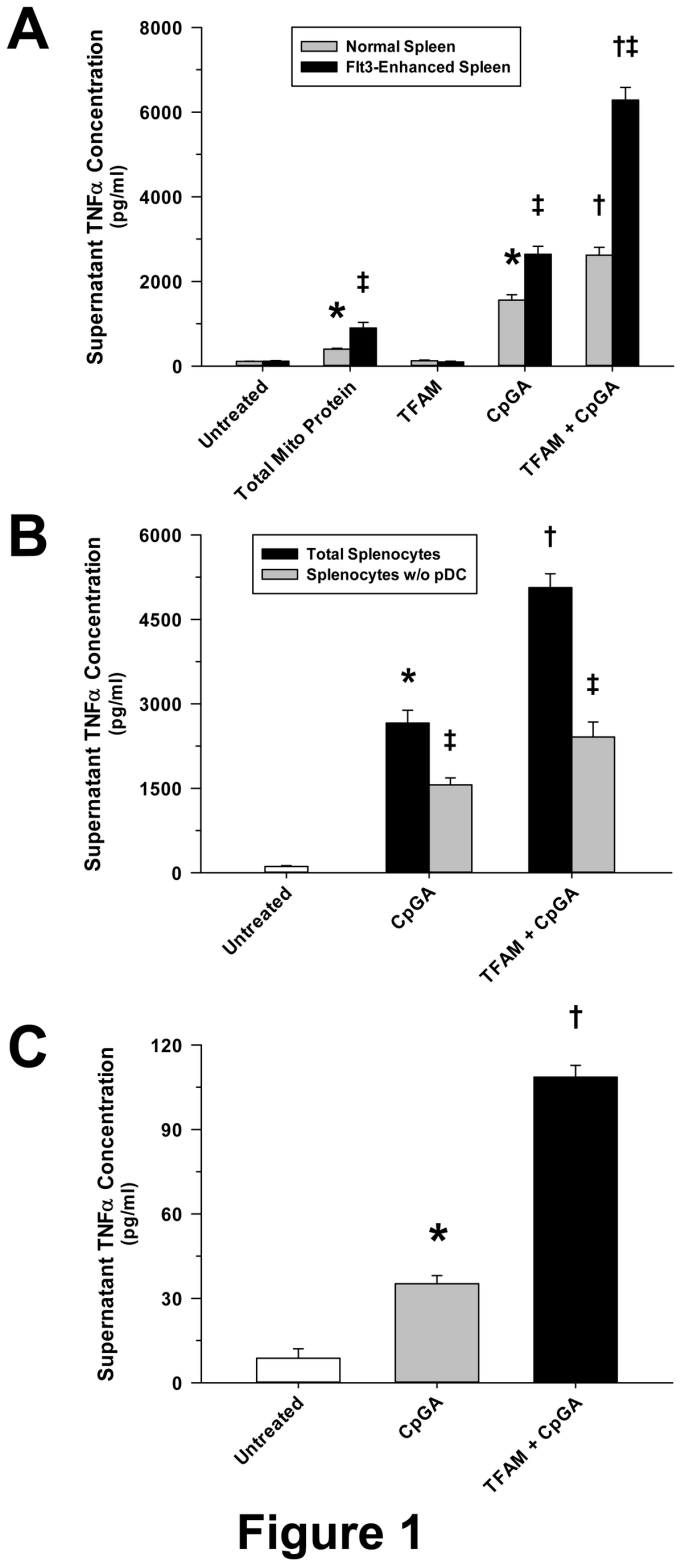
TFAM Augments CpGA DNA-induced Splenocyte TNFα Release which Is Highly Dependent upon the Presence of Plasmacytoid Dendritic Cells. The presented data was derived from at least 5 independent experiments. (A) As previously observed for other cytokines in different cell preparations [2,3], native mitochondrial protein (5 µg/ml) induced significant TNFα release from cultured mouse Flt3L-expanded splenocytes (1 × 10^6^ cells/ml) 24 hours post-treatment. CpGA DNA (0.3 µM), which is akin to that naturally associated with mitochondrial protein, promoted splenocyte TNFα release which was significantly amplified by co-incubation with recombinant human TFAM (5 µg/ml) despite that having no effect when treated alone. Compared to splenocytes obtained from a normal spleen, TNFα release was markedly increased following all treatments when the constituent pDC population was expanded by Flt3L over-expression as employed in the present study. (B) Selective removal of pDCs from matching Flt3L-expanded splenocyte preparations notably reduced the TNFα release induced by CpGA DNA (0.3 µM) ± TFAM (5 µg/ml) 24 hours post-treatment. (C) pDCs (1 × 10^6^ cells/ml) isolated from similar mouse splenocyte preparations demonstrated comparably minimal yet significant TNFα release 24 hours after CpGA DNA (0.3 µM) ± TFAM (5 µg/ml) treatment (*p < 0.01, relative to no treatment; ^†^ *p* < 0.01, compared to CpGA DNA treatment alone, and ^‡^ *p* < 0.01, relative to matching normal splenocyte treatments).

In keeping with our previous investigations demonstrating pDC Type I IFN release [2] and peripheral blood monocyte IL-8 release [3] in response to mitochondrial danger signals, mitochondrial protein isolated from HepG2 cells induced significant TNFα release in cultured Flt3L-expanded mouse splenocytes (Figure 1A). Moreover, as previously observed with DCs and monocytes [2,3,13], treatment with highly-purified recombinant human TFAM alone did not stimulate cytokine release. However, TFAM significantly amplified TNFα release in response to CpGA DNA treatment. For the sake of comparison, this effect was first demonstrated in splenocytes obtained from a normal spleen. In the present study, these results were further augmented following DC expansion consequent to Flt3L over-expression (Figure 1A) while being mitigated following the depletion of pDCs from matching splenocyte preparations (Figure 1B). Finally, co-treatment of TFAM with CpGA DNA was observed to promote greater TNFα release as well from isolated pDCs compared to CpGA DNA alone (Figure 1C), albeit comparatively much less overall than that released from treated splenocyte preparations.

### Splenocyte TNFα release in response to TFAM and CpGA DNA was dependent upon RAGE and TLR9 receptors

**Figure 2:**
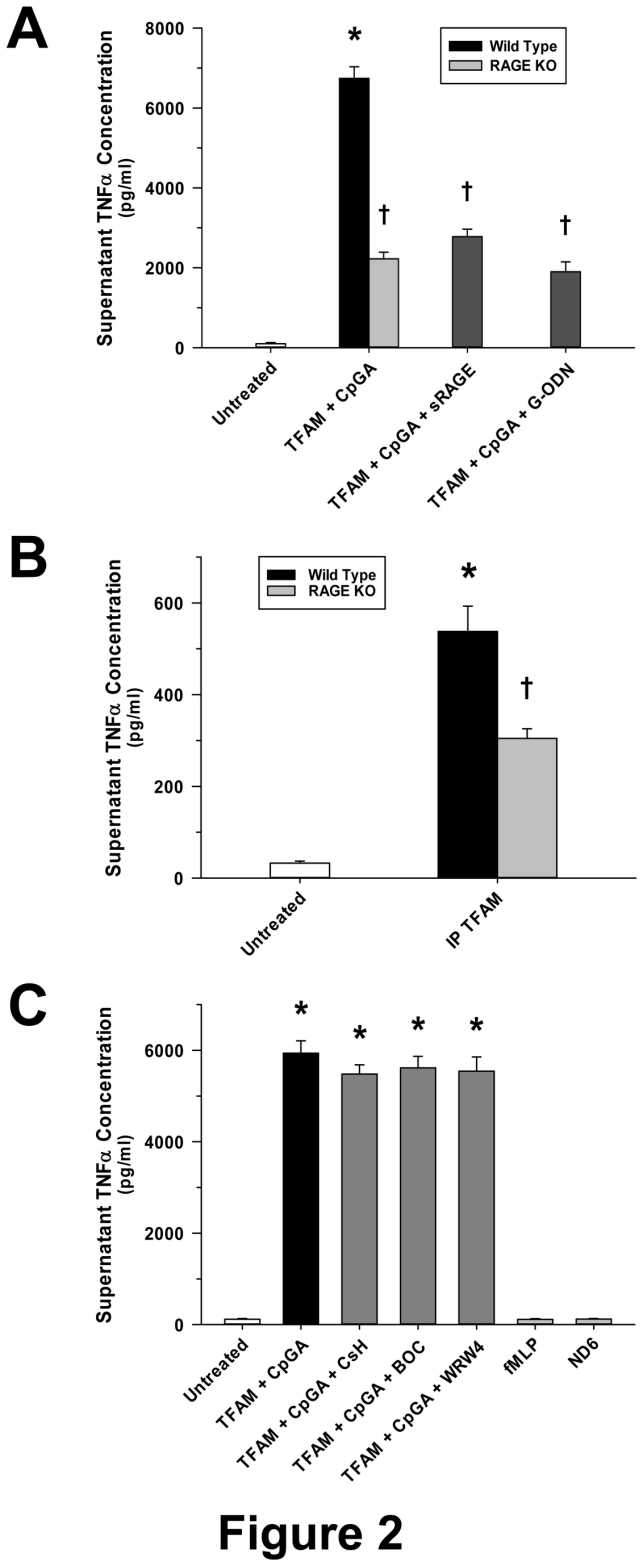
Amplification of CpGA DNA-provoked Splenocyte TNFα Release by TFAM Involves RAGE and TLR9 but Not FPR. The presented data was derived from at least 5 independent experiments. (A) 24 hours post-treatment, TFAM (5 µg/ml) increased CpGA DNA (0.3 µM)-induced Flt3L-expanded mouse splenocyte (1 × 10^6^ cells/ml) TNFα release. This effect was dramatically attenuated through RAGE (sRAGE, 20 µg/ml) or TLR9 (G-ODN, 10 µM) inhibition 30 minutes pre-treatment. In addition, splenocytes from RAGE -/- mice demonstrated significantly diminished TNFα release in response to TFAM ± CpGA DNA treatment. (B) Similarly, exposure of Flt3L-expanded splenocytes (1 × 10^6^ cells/ml) to immunoprecipitated native TFAM complexed with mtDNA (IP TFAM, 0.5 µg/ml) derived from necrotic cells yielded marked release of TNFα by 24 hours post-treatment which was significantly reduced when treating corresponding splenocytes from RAGE -/- mice. (C) Pre-treatment (30 minutes) with FPR antagonists [CsH (1 µM), BOC (10 µM) or WRW4 (20 µg/ml)] had no effect upon TFAM (5 µg/ml) + CpGA DNA (0.3 µM)-induced splenocyte TNFα release 24 hours post-treatment. Likewise, 24 hours after treatment, FPR agonists [fMLP (5 µg/ml), ND6 (5 µg/ml)] did not stimulate TNFα release (*p < 0.01, compared to no treatment; ^†^ *p* < 0.01, relative to the wild type TFAM + CpGA treatment group).

As expected, CpGA DNA-dependent TNFα release from Flt3L-expanded mouse splenocyte preparations was reliant upon TLR9, as reflected by attenuation of the response by the TLR9-blocking oligonucleotide, G-ODN (Figure 2A). TFAM binds with high affinity to RAGE to promote cell migration [14] and was previously shown to amplify IFNα release in splenocyte cultures [2]. The present results demonstrated that the augmentation of CpGA DNA-induced TNFα release by TFAM and the response to the TFAM-mtDNA complex (IP TFAM) derived from necrotic cells was dependent upon RAGE (Figure 2A and 2B, respectively). In contrast, the contribution of *N*-formylated mitochondrial proteins, which are recognized by formyl peptide receptors and are potentially immunogenic [3,15,16], was shown to be nil in cultured mouse splenocytes (Figure 2C).

### Immune cell recognition of TFAM is charge-dependent and is equivalent for TFAM DNA-binding subunit proteins Box 1 and Box 2

**Figure 3:**
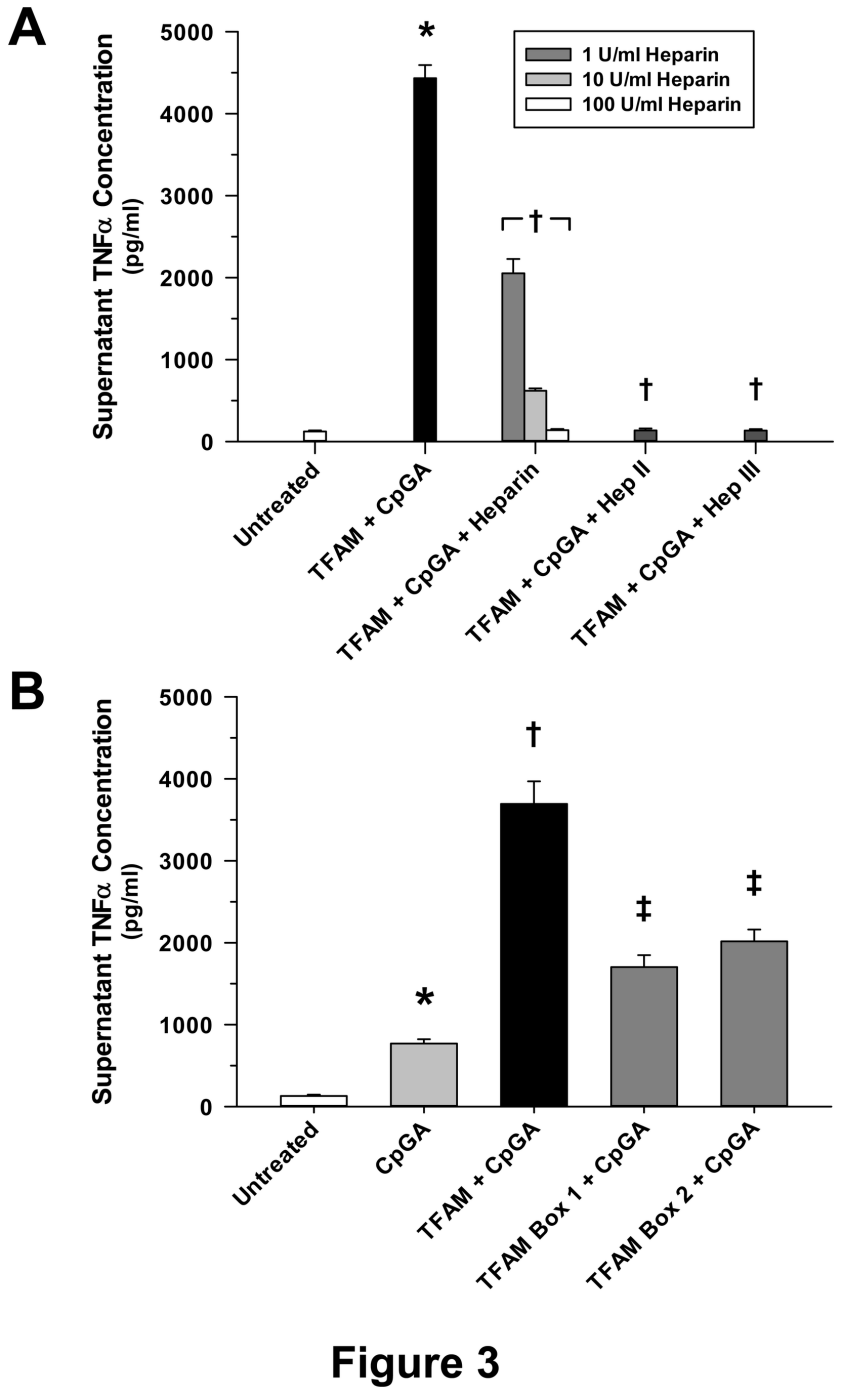
Enhancement by TFAM of the CpGA DNA-induced Splenocyte Proinflammatory Immune Response Results from Charge-Dependent Recognition of TFAM and Is Equipotent for Both TFAM DNA-binding Subunit Proteins, Box 1 and Box 2. The presented data was derived from at least 5 independent experiments. (A) Pre-treatment (30 minutes) with heparin sodium (1-100 U/ml) or heparin lyase (heparinase) II or III (5 mU/ml) dramatically suppressed TFAM (5 µg/ml) + CpGA DNA (0.3 µM)-induced Flt3L-expanded splenocyte (1 × 10^6^ cells/ml) TNFα release 24 hours post-treatment. Heparin demonstrated significant inhibition in a dose-dependent manner (*p < 0.01, compared to no treatment; † *p* < 0.01, relative to the TFAM + CpGA treatment group). (B) Though not as potent at augmenting CpGA DNA (0.3 µM)-induced splenocyte (1 × 10^6^ cells/ml) TNFα release as full-length recombinant TFAM (5 µg/ml) 24 hours post-treatment, recombinant TFAM Box 1 and Box 2 (5 µg/ml) each demonstrated significant amplification of CpGA DNA-induced TNFα release (*p < 0.01, compared to no treatment; † *p* < 0.01, relative to CpGA DNA treatment alone, and ‡*p* < 0.01, compared to CpGA DNA alone and the TFAM + CpGA treatment group).

Previous studies have shown that HMG proteins have high affinity for heparin sulfate moieties located on the cell surface, and heparin sulfate binding is necessary for RAGE-dependent signaling. Heparin, which possesses binding properties similar to heparin sulfate, is a competitive inhibitor of RAGE signaling [17]. These experiments demonstrated dose-dependent inhibition of the immunogenic properties of TFAM-CpGA DNA complexes by heparin (Figure 3A). Similarly, heparin lyases, which remove heparin sulfate moieties from the cell surface [17], completely attenuated Flt3L-expanded mouse splenocyte TNFα release in response to TFAM + CpGA DNA (Figure 3A). In keeping with charge-dependent, rather than sequence-dependent recognition of TFAM by immune cells, recombinant human TFAM Box 1 and Box 2 HMG protein motifs were equally capable of enhancing CpGA DNA-induced TNFα release in cultured Flt3L-expanded mouse splenocytes (Figure 3B). As with intact full-length TFAM, treatment with recombinant TFAM Box 1 and Box 2 proteins alone did not promote TNFα release, and heparin competitively inhibited their proinflammatory actions (*data not shown*).

### TFAM-mediated TNFα release was dependent upon endosomal processing, including DNA binding, endosomal acidification and TLR9 cleavage

**Figure 4:**
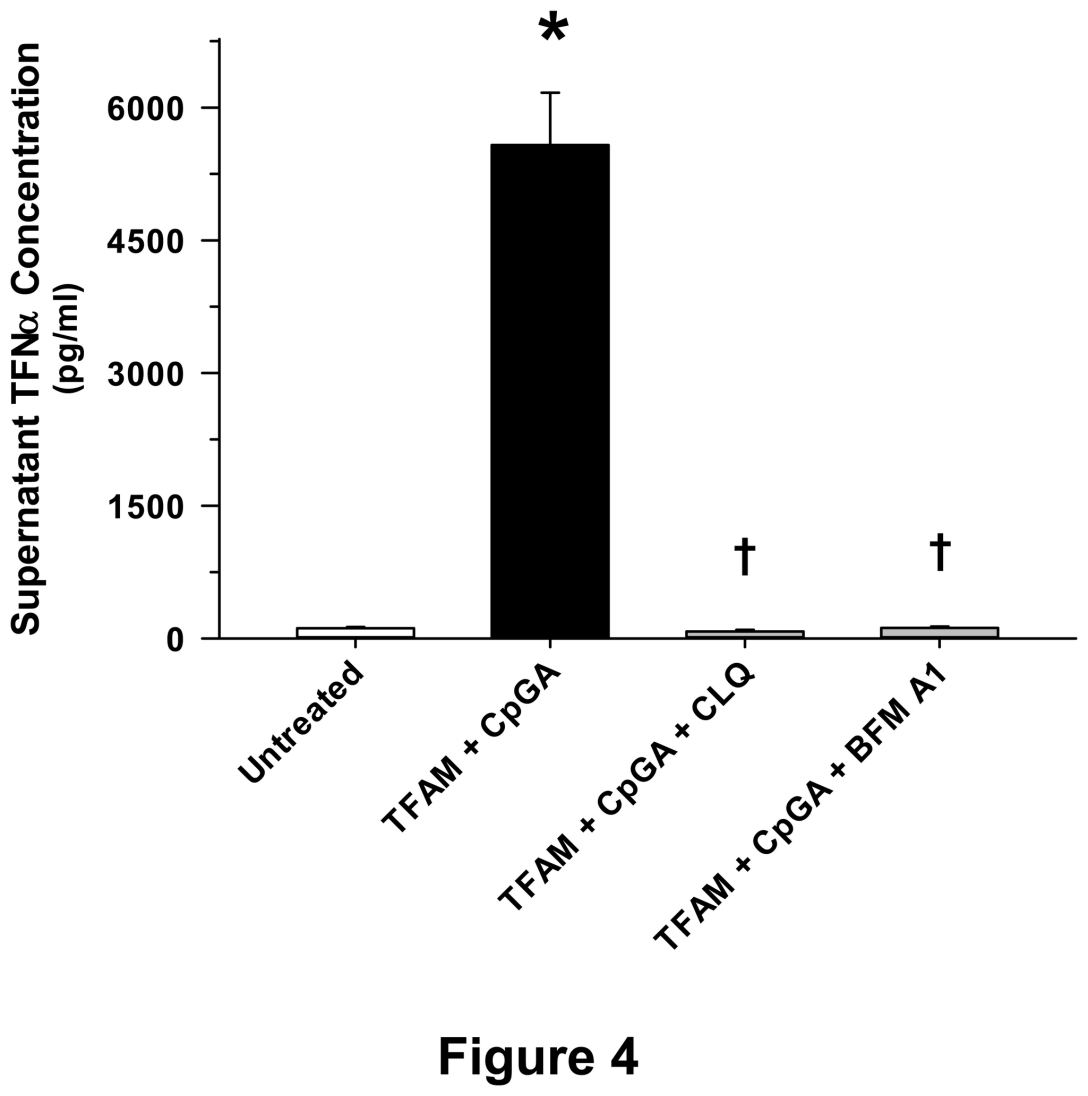
TFAM-CpGA DNA Complexes Produce a Proinflammatory Response in Splenocytes which Is Dependent upon Endosomal Processing. The presented data was derived from at least 5 independent experiments. TFAM (5 µg/ml) + CpGA DNA (0.3 µM)-induced Flt3L-expanded splenocyte (1 × 10^6^ cells/ml) TNFα release was completely prevented 24 hours post-treatment by inhibiting nucleic acid binding to TLR9 or endosomal acidification with chloroquine (CLQ, 10 µM) or Bafilomycin A1 (BFM A1, 1 µM) pre-treatment (30 minutes) respectively (*p < 0.01, relative to no treatment; † *p* < 0.01, compared to the TFAM + CpGA treatment group).

**Figure 5:**
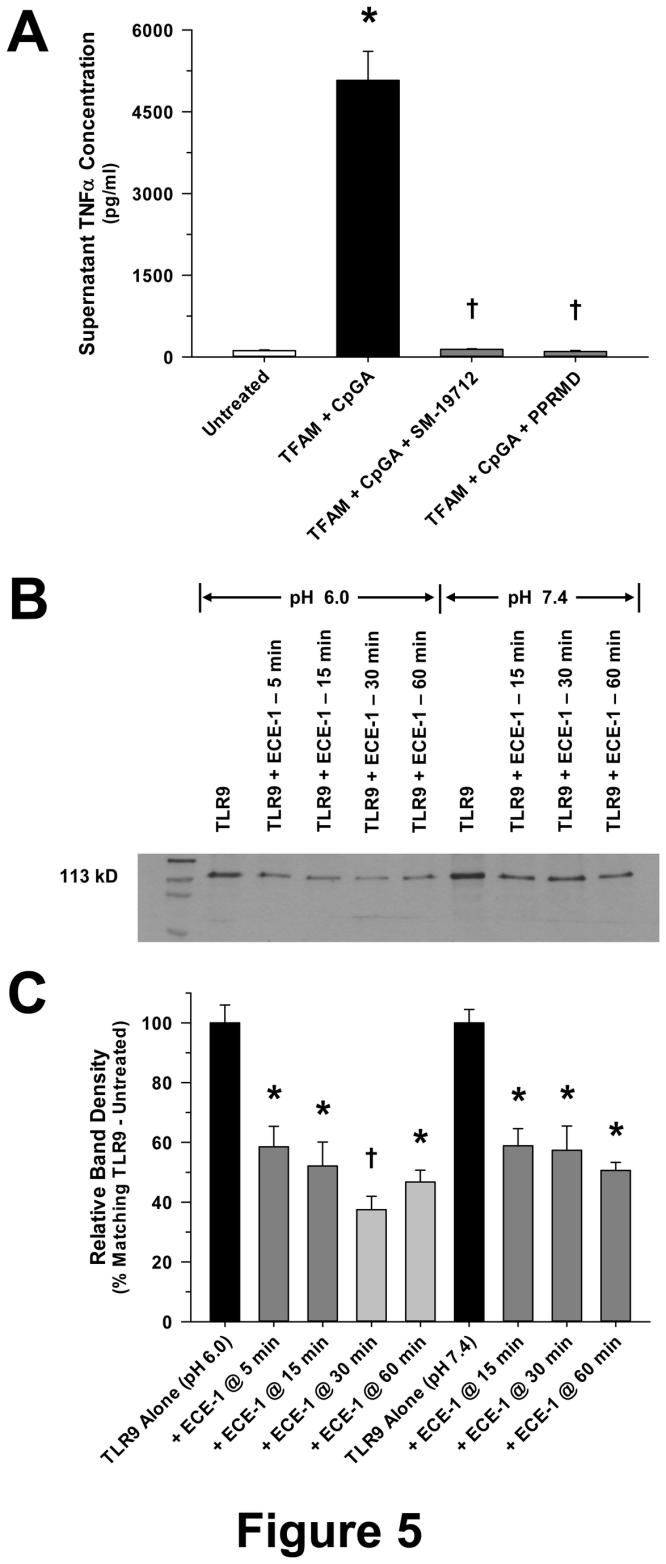
Inhibition of Endosomal Receptor Recycling Prevents TFAM + CpGA DNA-induced Splenocyte TNFα Release. (A) The presented data was derived from at least 5 independent experiments. Pre-treatment (30 minutes) of Flt3L-expanded splenocytes (1 × 10^6^ cells/ml) with inhibitors of endothelin converting enzyme-1 (ECE-1) (SM-19712, 10 µM) or phosphoramidon (PPRMD, 100 µM), which impede endosomal receptor recycling, totally prohibited TFAM (5 µg/ml) + CpGA DNA (0.3 µM)-induced TNFα release 24 hours post-treatment (*p < 0.01, relative to no treatment; † *p* < 0.01, compared to the TFAM + CpGA treatment group). (B) Representative photomicrograph of a Western blot showing degradation of TLR9 (50 µM) over time by *in vitro* incubation with ECE-1 (0.3 µg/ml) under optimal (6.0) and normal (7.4) pH conditions. (C) The presented data is representative of at least 3 independent experiments. Relative band expression of TLR9 was dramatically reduced at all time-points and under both assay pH conditions following co-incubation with ECE-1. As expected, evident degradation appeared more marked under optimal conditions (*p < 0.05, relative to matching TLR9 Alone treatment group; † *p* < 0.05, compared to matching TLR9 Alone and TLR9 + ECE-1 @ 5 min treatment groups).

TLR9-mediated inflammatory responses depend upon endosomal recognition of DNA [2,18] and cleavage of TLR9 [19]. However, uncertainty exists in terms of the enzyme(s) responsible for TLR9 cleavage and activation [20]. In keeping with known mechanisms of TLR9 signaling, chloroquine, an inhibitor of nucleic acid binding to TLR9 [21], and Bafilomycin A1, an inhibitor of endosomal acidification [22], completely suppressed Flt3L-expanded splenocyte TNFα production in response to TFAM and CpGA DNA treatment (Figure 4). Based upon recent studies showing that endothelin converting enzyme-1 (ECE-1) is capable of promoting the endosomal recycling and reactivation of other receptors [23,24], we sought to determine whether ECE-1 played a role in TLR9 signaling. Recognizing the presence of putative hydrophobic binding sites within TLR9 [e.g., sequences 446-447 and 886-887 of the full-length (1032 amino acids) human TLR9 protein (http://www.ncbi.nlm.nih.gov/protein/AAZ95519.1)] and considering that ECE-1 is pH-sensitive [23], we further tested the hypothesis that ECE-1 cleaves TLR9, particularly at low pH. ECE-1 was shown to be essential for induction of TNFα release by TFAM-CpGA DNA, as reflected by the potent effects of the specific ECE-1 inhibitor, SM-19712, and the non-specific inhibitor of this class of enzyme, phosphoramidon (PPRMD) (Figure 5A). Furthermore, ECE-1 was observed to rapidly cleave TLR9 (within 5 minutes) and did so more potently at lower physiological pH, modeling conditions in mature endosomes (Figure 5B and 5C). To our knowledge, this is the first study to demonstrate that ECE-1 cleaves TLR9 and regulates TLR9-mediated cytokine release.

### Sequential signal transduction through PI3K and NF-κB is required for TNFα release from splenocytes in response to TFAM and CpGA DNA

**Figure 6:**
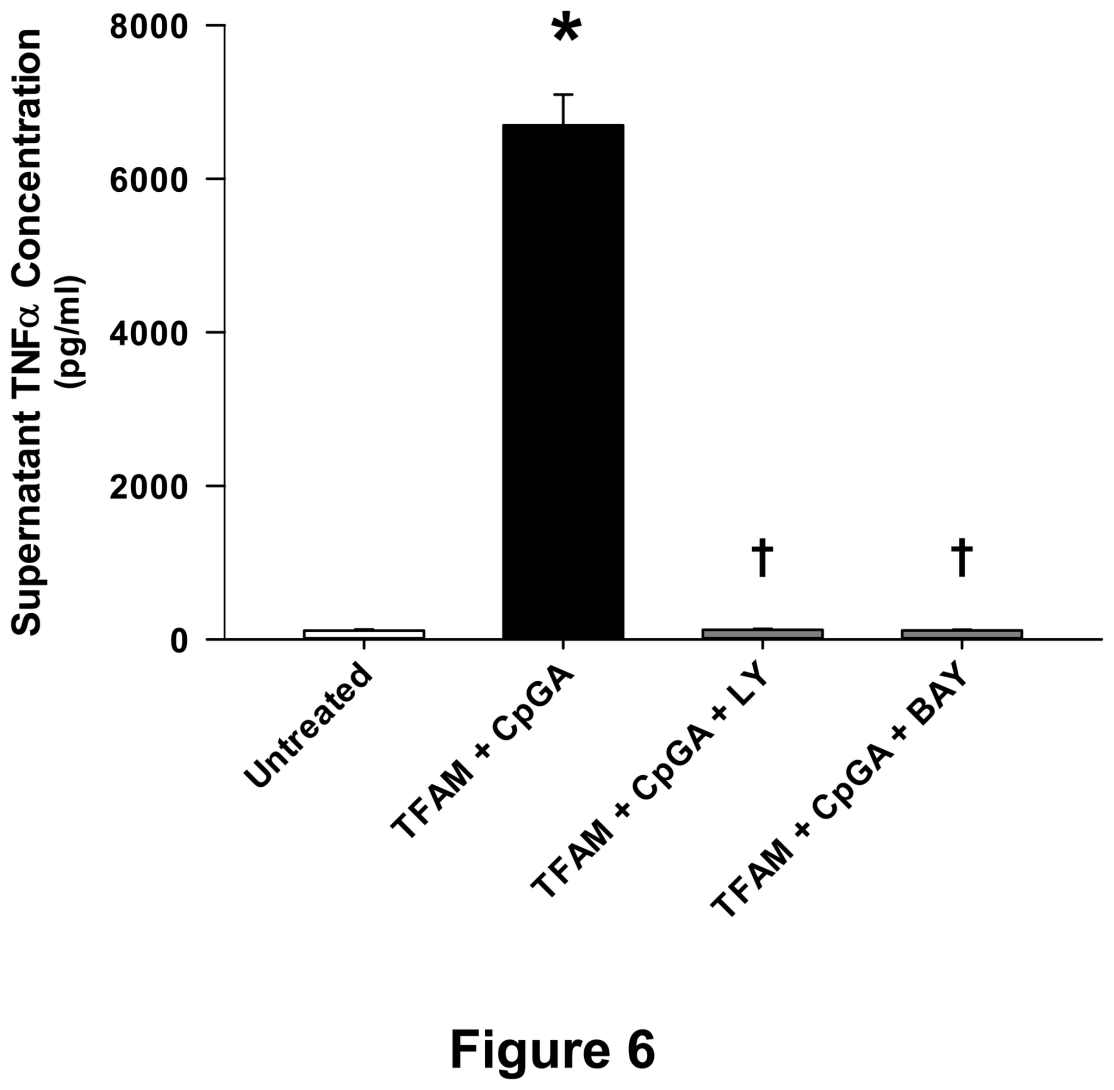
TFAM and CpGA DNA Induce a Splenocyte Proinflammatory Immune Response through PI3K and NF-κB Signaling. The presented data was derived from at least 5 independent experiments. TFAM (5 µg/ml) + CpGA DNA (0.3 µM)-induced Flt3L-expanded splenocyte (1 × 10^6^ cells/ml) TNFα release was completely blocked 24 hours post-treatment following pre-treatment (30 minutes) with inhibitors of PI3K (LY294002, 5 µM) and NF-κB (BAY 11-7085, 5 µM) signaling (*p < 0.01, relative to no treatment; † *p* < 0.01, compared to the TFAM + CpGA treatment group).

**Figure 7:**
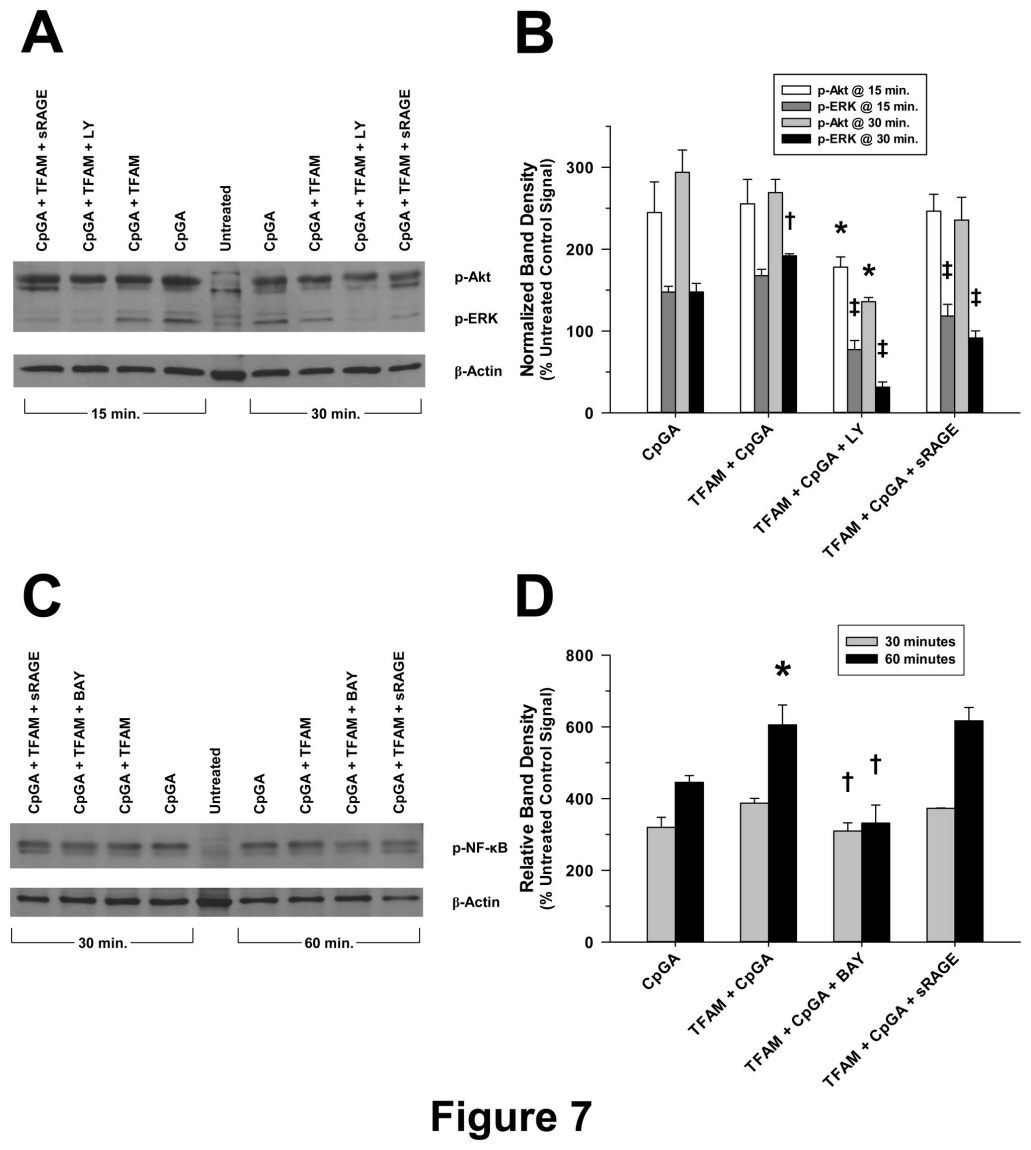
Cell Signaling in Mouse Splenocytes in Response to TFAM + CpGA DNA Exposure Involves the Akt, ERK and NF-κB Pathways. The presented data is representative of at least 3 independent experiments. (A) Representative photomicrograph of a Western blot demonstrating the changes observed in p-Akt and p-ERK signaling 15 and 30 minutes after CpGA DNA alone or in combination with TFAM treatment and the latter following LY294002 or sRAGE pre-treatment. (B) Relative band expression of each protein was dramatically elevated in response to both treatments at each time-point and markedly reduced by PI3K (LY) inhibition. RAGE inhibition (sRAGE) diminished p-ERK only (*p < 0.01, relative to the matching p-Akt signal induced by TFAM + CpGA treatment; † *p* < 0.01, compared to the corresponding CpGA DNA treatment alone, and ‡*p* < 0.01, relative to the matching p-ERK signal induced by TFAM + CpGA treatment). (C) Representative photomicrograph of a Western blot demonstrating the changes observed in p-NF-κB signaling 30 and 60 minutes after CpGA DNA alone or in combination with TFAM treatment and the latter following BAY 11-7085 or sRAGE pre-treatment. (D) Relative band expression was notably increased in response to both treatments at each time-point and decreased by NF-κB (BAY) inhibition. RAGE inhibition (sRAGE) had no discernable effect (*p < 0.01, compared to the corresponding CpGA DNA treatment alone; † *p* < 0.01, relative to the matching signals induced by TFAM + CpGA treatment). In both cases, β-actin served as a loading control for normalization.

As illustrated previously in the context of Type I interferon responses [2], TNFα release in response to TFAM-CpGA DNA complexes was dependent upon PI3K and NF-κB signaling, as reflected by the inhibitory effects of LY294002 and BAY 11-7085, respectively (Figures 6 and 7). As previously shown, the Akt and ERK signaling pathways were co-regulated by PI3K (Figure 7A and 7B) [2]. Of note, pharmacological inhibition of ERK (using the MEK1/2 inhibitor, U0126) only partially inhibited TNFα release under these experimental conditions (*data not shown*). Furthermore, the sequential timing of signal pathway activation followed the pattern of early (within 15 minutes) Akt phosphorylation, then ERK and NF-κB phosphorylation, peaking at 30 and 60 minutes, respectively (Figure 7).

## Discussion

This study confirms an important role of TLR9-expressing pDCs in the induction of proinflammatory immune responses to mitochondrial danger signals, particularly TFAM and CpG-enriched (mitochondrial) DNA. The *ex vivo* Flt3L-expanded splenocyte preparation employed herein is highly relevant as the model recapitulates *in vivo* immune responses to infectious and non-infectious (sterile) danger signals [25,26]. In this model, it was shown that pDCs, which are present in low abundance (~3%) [2], greatly amplified the production of TNFα in response to TFAM and CpG DNA via “bystander” activation [8] of other immune cells in a model representing more complex *in vivo* immune responses (i.e., native immune cell populations represented by splenocyte cultures). This clearly implies that these specialized cells play an important role in the setting of sterile inflammatory responses. The data further indicated that the signaling pathways regulating TNFα release in this context are identical to those promoting Type I IFNα release [2] and that pDCs serve to significantly amplify TNFα release. The simultaneous release of TNFα and Type I IFNs through pDC activation has interesting implications, as these cytokines are hypothesized to counteract each other in the setting of sterile inflammation, and imbalances between these cytokines are believed to contribute to a spectrum of acute and chronic human diseases [27]. As such, this study suggests that pDC responses to TFAM and mtDNA, which are released in the setting of cell damage [2,3,28], could be critical for the regulation of sterile immune responses in the setting of inflammatory and autoimmune diseases [27].

While other immune cell receptors and mitochondrial danger signals have been implicated in the pathogenesis of sterile inflammation [29], it was evident from these studies that RAGE facilitates the immunogenic response to TFAM and CpGA DNA. In contrast, formyl peptide receptors, which are shown to promote proinflammatory responses to other mitochondrial components (i.e., *N*-formylated components of the cytochrome complexes [3]), did not contribute to the observed TNFα response to TFAM and CpGA DNA (Figure 2C). Unlike TLRs, which bind with some specificity to molecular epitopes (e.g., CpG motifs), RAGE recognizes highly charged molecules, including glycated proteins, HMGB1 and TFAM, through heparin sulfate moieties [17,30]. As such, RAGE-dependent signaling was competitively inhibited by pre-treatment with highly charged molecules (heparin) or by removing the heparin sulfate moieties (via heparin lyase pre-treatment). Unlike TLR9, which is located intracellularly and engages antigens within endosomes [13], RAGE is a transmembrane receptor that facilitates antigen transport to endosomes [31]. The observed inhibition of TNFα release from Flt3L-expanded splenocytes by sRAGE, heparin and heparin lyase pre-treatment, and in splenocyte preps from RAGE -/- mice, indicated that RAGE participated in the sensing of CpGA DNA, and that TFAM enhanced RAGE-dependent inflammation (Figures 2A and 3A). Given that TFAM closely resembles HMGB1 in structure and function [32] and that the latter is a well-known RAGE agonist [17,31], our results are not unexpected. However, it should be noted that TFAM, not HMGB1, is tightly bound to mtDNA (e.g., they remain associated when TFAM is immunoprecipitated from mitochondrial preparations [2]). Thus, focusing on TFAM as a critical danger signal in the setting of sterile inflammation remains justified.

Like HMGB1, TFAM possesses two HMG DNA-binding regions or “Box” proteins connected by a short bridging sequence, resulting in an L-shaped tertiary structure that effectively binds and bends DNA to facilitate transcription and repair [33]. Based upon previous studies with HMGB1 showing that the two HMG Box sequences have contrasting immunogenic properties, namely, HMGB1 Box A suppresses whereas Box B induces inflammation [34], we sought to determine whether the same was true for TFAM Box 1 and Box 2 sequences. As presented in Figure 3B, TFAM Box 1 and Box 2 proteins were equivalent in their capacities to promote TNFα release by CpGA DNA. Furthermore, and unlike HMGB1 wherein a specific 20 amino acid sequence is shown to be responsible for the immunogenicity of Box B protein [35], the proinflammatory properties of TFAM Box 1 and Box 2 are likely dependent upon molecular surface charge characteristics [36], as reflected by the competitive inhibition by heparin. Considering that TFAM is known to stabilize DNA [33] and based upon recent studies suggesting that HMG peptides enhance TLR9 binding to the cleaved form of TLR9 to promote more effective signaling [37], TFAM likely enhances TLR9 signaling through multiple mechanisms.

While RAGE serves to significantly amplify the immune response to TFAM-CpGA DNA complexes, putative TLR9 signaling pathways were shown to be essential for TNFα release. In keeping with a TLR9-dependent immune response, G-ODN, a TLR9-blocking oligonucleotide, chloroquine, a potent inhibitor of nucleic acid binding to TLR9 within endosomes [38], and Bafilomycin A, an inhibitor of endosomal acidification [22], all attenuated the immune response to TFAM and CpGA DNA (Figures 2A and 4 respectively). Additionally, inhibition of ECE-1, a metalloprotease that cleaves ligands from endosomal receptors to promote receptor recycling and reactivation [23], prevented the immune response to TFAM and CpG DNA (Figure 5A). In this regard, it is known that cleavage of TLR9 in acidified endosomes is essential for TLR9-dependent immune responses [19]. While pH-sensitive cathepsins have been incriminated as potential mediators of TLR9 cleavage, recent studies confirm that cathepsins are not essential for TLR9 activation, leaving the question open as to which enzyme(s) are necessary for TLR9 activation [39]. Furthermore, the mechanisms of TLR9 activation are shown to differ in various immune cell lines [20]. Previous investigations indicate that ECE-1, a pH-sensitive metalloprotease localized to endosomes, cleaves antigens to facilitate recycling (reactivation) of endosomal receptors other than TLR9 [23,24]. Our results provide evidence that ECE-1 can cleave and activate TLR9. Specifically, ECE-1 was observed to cleave recombinant TLR9, particularly at lower physiological pH, such as exists in mature endosomes (Figure 5B and 5C). Using a potent inhibitor of metalloproteases (phosphoramidon) and a highly specific inhibitor of ECE-1 (SM-19712) [40,41], we observed complete inhibition of TFAM-CpGA DNA (TLR9)-mediated TNFα release from splenocytes (Figure 5A). Mechanistically, cleavage of TLR9 by ECE-1 may serve to enhance DNA binding to TFAM-CpGA DNA [20,37], could facilitate TLR9 recycling and reactivation [42], and could further regulate the duration of the immune response by influencing the rate of TLR9 degradation [43]. Additional studies are required to clarify the mechanisms by which ECE-1 participates in TLR9 signaling.

**Figure 8:**
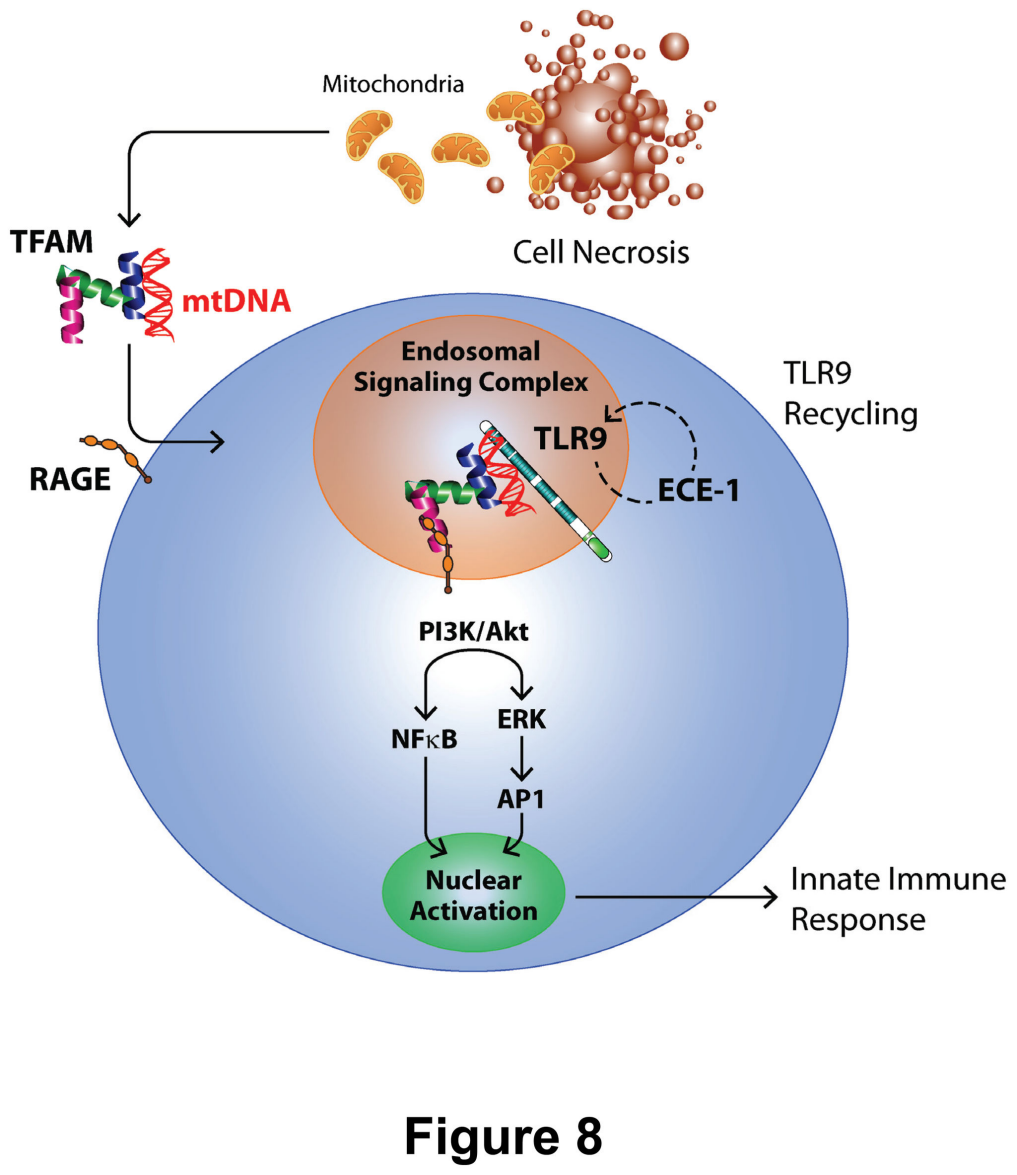
Schematic Representation of Splenic Dendritic Cell Activation by Necrotic Cells. Mitochondrial DNA (mtDNA), when released from necrotic cells, remains associated with TFAM. As in Type I interferon production, TFAM augments the proinflammatory immune response to mtDNA by associating with RAGE (and related heparin sulfate moieties) and TLR9, respectively, to promote endosomal processing, including endosomal receptor (TLR9) recycling by ECE-1, and signal transduction via PI3K/Akt and ERK pathway activation to induce splenic dendritic cell TNFα gene transcription and protein translation and release.

In addition to the aforementioned endosomal mechanisms, and in keeping with established signaling events required for Type I IFN production in response to TFAM-DNA complexes [2], the release of TNFα was shown to depend upon PI3K and NF-κB signaling pathways (Figures 6 and 7). Our results indicated sequential phosphorylation of Akt, ERK and NF-κB following activation by TFAM and immunogenic DNA, represented schematically in Figure 8. As such, the production of TNFα and Type I IFNs occur through common signaling pathways [2,18]. It is interesting to note that sRAGE significantly decreased TFAM + CpGA DNA-induced TNFα release (Figure 2A) but did not influence NF-κB signaling (Figure 7D), suggesting that RAGE augments TNFα release through other, as yet to be determined, signaling pathways. One could surmise that RAGE may serve to stabilize the activation of NF-κB over time (e.g., by promoting endosomal signal complex formation with TLR9) or could activate other signaling pathways that serve to amplify the effects of NF-κB (e.g., ERK-related activation of transcription factor AP-1), but further investigation would be needed to clarify this mechanism. The simultaneous induction of proinflammatory (TNFα) and potentially counter-regulatory (Type I IFNs) cytokines by pDCs in response to mitochondrial danger signals is consistent with previous studies showing that NF-κB co-regulates the expression of Type I IFNs and TNFα through the nuclear transcription factors, IRF-7 and IRF-5, respectively [6]. Our findings demonstrated that pDCs are proximal regulators of the sterile immune response, and we speculate that pDCs could influence the observed clinical variability of sterile immune responses, including systemic inflammation and autoimmunity [44–46], by influencing the balance of Type I IFN and TNFα [27,42].

As expected, there are certain limitations to animal models or investigations involving cells localized to one tissue compartment. While the Flt3L-expanded splenocyte model employed herein may not reflect immune responses in other tissue compartments, the spleen is recognized to be a major source of TNFα during systemic illnesses caused by sterile inflammation [47,48] and in infectious models of systemic inflammation [49]. Notably, the splenocyte preparation responds vigorously to TFAM and associated mtDNA derived from necrotic cells (Figure 2B). As such, the spleen is a likely source of inflammatory cytokine release in the setting of systemic mitochondrial danger signal release [1]. The pDC depletion protocol does not account for immature myeloid DCs, which are also capable of responding to TLR9 ligands [50], and this likely explains why depletion of pDCs did not completely attenuate the response to mitochondrial antigens or to CpGA DNA ± TFAM (Figure 1B). It is further unclear how proinflammatory and regulatory immune responses interact or whether the net balance between the two is modified by TFAM. Further clarification of these important mechanisms will require additional research.

In conclusion, this study indicated that pDCs promote an acute proinflammatory immune response to mitochondrial danger signals, particularly TFAM and CpG-enriched (mitochondrial) DNA, and that pDCs greatly amplify the innate immune response through “bystander” activation in a splenocyte culture model representing complex interactions *in vivo*. While purified TFAM is non-immunogenic, when coupled with DNA, it was demonstrated, as shown schematically in Figure 8, to augment TNFα release through RAGE, TLR9 and related signaling pathways, including endosomal processing and activation of PI3K/Akt, ERK and NF-κB. In this regard, to our knowledge this study is the first to identify ECE-1, and presumably TLR9 cleavage by this enzyme, as a critical step in TLR9 signaling. Unlike its homologue HMGB1, which possesses distinct anti-inflammatory (Box A) and proinflammatory (Box B) protein sequences [34], TFAM Box 1 and Box 2 proteins were shown to be equipotent in terms of their immunogenic properties. Inhibition with heparin and heparin lyases indicated that heparin sulfate moieties are essential for the recognition of TFAM and DNA, which could explain, in part, the protective effects of heparin in the context of acute inflammation [51,52]. Hence, the results of these investigations will provide focus for future studies seeking to mitigate the potentially harmful innate immune response occurring in the context of acute and chronic tissue damage.
